# Clinical Biomarkers for Early Identification of Patients with Intracranial Metastatic Disease

**DOI:** 10.3390/cancers13235973

**Published:** 2021-11-27

**Authors:** Karolina Gaebe, Alyssa Y. Li, Sunit Das

**Affiliations:** 1 Institute of Medical Science, Faculty of Medicine, University of Toronto, 1 King’s College Circle, Toronto, ON M5S 3K1, Canada; karolina.gaebe@mail.utoronto.ca (K.G.); alyssa.li@mail.utoronto.ca (A.Y.L.); 2 Division of Neurosurgery, St. Michael’s Hospital, University of Toronto, 30 Bond Street, Toronto, ON M5B 1W8, Canada

**Keywords:** brain metastases, intracranial metastatic disease, biomarker, non-small cell lung cancer, breast cancer, melanoma

## Abstract

**Simple Summary:**

The development of brain metastases, or intracranial metastatic disease (IMD), is a serious and life-altering complication for many patients with cancer. While there have been substantial advancements in the treatments available for IMD and in our understanding of its pathogenesis, conventional methods remain insufficient to detect IMD at an early stage. In this review, we discuss current research on biomarkers specific to IMD. In particular, we highlight biomarkers that can be easily accessed via the bloodstream or cerebrospinal fluid, including circulating tumor cells and DNA, as well as advanced imaging techniques. The continued development of these assays could enable clinicians to detect IMD prior to the development of IMD-associated symptoms and ultimately improve patient prognosis and survival.

**Abstract:**

Nearly 30% of patients with cancer will develop intracranial metastatic disease (IMD), and more than half of these patients will die within a few months following their diagnosis. In light of the profound effect of IMD on survival and quality of life, there is significant interest in identifying biomarkers that could facilitate the early detection of IMD or identify patients with cancer who are at high IMD risk. In this review, we will highlight early efforts to identify biomarkers of IMD and consider avenues for future investigation.

## 1. The Burden of Intracranial Metastatic Disease

Intracranial metastatic disease (IMD) is a common complication of many primary cancers [1]. Although the precise incidence is unknown, IMD is estimated to occur in nearly 30% of patients with systemic malignancies with growing prevalence due to several factors, including prolonged survival, improved surveillance, and increasing population age [1]. Incidence rates may be even higher in patients with certain primary cancers, most notably non-small cell lung cancer (NSCLC), breast cancer, and melanoma, which account for 45%, 15%, and 10% of IMD in some studies [2,3,4]. A diagnosis with IMD substantially impacts the course of disease, reducing survival and quality of life, while increasing healthcare utilization and cost [5]. Conventional treatment options for IMD include surgical resection, stereotactic radiosurgery (SRS), and whole brain radiation therapy (WBRT) [6]. In order to help physicians guide treatment decisions and provide survival estimates for patients with IMD, prognostic classification systems based on age, performance status, and extracranial and intracranial disease burden have been developed [7,8]. Patients presenting with fewer lesions and good performance status typically receive neurosurgery or SRS, while patients with a higher number of intracranial lesions or poor performance status are primarily treated with WBRT [6]. Excitingly, recent data suggest that some patients with IMD may respond to systemic targeted therapies and immunotherapies, particularly patients with IMD secondary to NSCLC, breast cancer, and melanoma [9,10].

## 2. A Need for Novel Diagnostic Tools

Magnetic resonance imaging (MRI) is currently the preferred modality for establishing a diagnosis of IMD [11]. MRI scans are now often conducted as part of the initial cancer staging work-up and, as a result, there has been an increase in the number of patients found to have asymptomatic IMD at initial cancer diagnosis [12]. IMD is otherwise typically identified by imaging prompted by the development of neurological symptoms. For this reason, current methods to identify IMD (using conventional imaging) often surrender any aim to detect IMD in its early stages, a shortcoming that is becoming increasingly important as the life expectancy of cancer patients increases [13]. Late diagnosis of IMD limits treatment options and aggravates symptom severity. Most imaging techniques are also unable to distinguish IMD from other radiographic findings that may present following treatment, such as pseudo-progression and radiation necrosis, and cannot differentiate between primary brain tumors and metastatic lesions [12,14]. There is a critical need to develop methods to detect early-stage IMD and to identify patients at high risk for IMD. Here, we will review avenues in development that hold promise, focusing on serum markers of neural tissue damage, genetic alterations acquired during the development of IMD, and imaging markers for IMD. 

## 3. Markers of Neurologic Damage in the Context of Intracranial Metastatic Disease

### 3.1. Neurofilament Light Chain

Neurofilament light chain (NfL) has been identified as a robust serum marker of autoimmune disease, traumatic brain injury, and neurodegenerative insult [15,16,17]. It is highly expressed in myelinated subcortical white matter axons and released into cerebrospinal fluid (CSF) or circulation as a result of neuronal decay [18]. Recent work by Winther-Larsen and colleagues suggests that NfL may present a potential diagnostic tool for IMD in patients with NSCLC. Serum NfL levels were comparable between patients with stage I NSCLC and stage IV NSCLC without IMD (16 pg/mL and 20 pg/mL, respectively, *p* = 0.702), implying that disease stage does not influence serum NfL concentrations. On the other hand, median NfL levels were significantly elevated in patients with stage IV NSCLC and IMD (34 pg/mL) compared to patients with stage I NSCLC (*p* = 0.002) and stage IV NSCLC without brain involvement (*p* = 0.015) [19]. Similarly, a cross-sectional analysis by Hepner et al. revealed serum NfL levels that varied closely with the presence and activity of intracranial disease in patients with primary central nervous system (CNS) tumors or IMD secondary to metastatic solid tumors. Among patients with active metastatic cancer, those with IMD had significantly higher serum NfL levels than those without IMD (161.7 pg/mL vs. 11.8 pg/mL, *p* = 0.0004). Both groups demonstrated significantly higher serum NfL levels than healthy adult controls (7.2 pg/mL) [20]. Darlix et al. also demonstrated significantly higher median serum levels of NfL in patients with breast cancer metastatic to the brain compared to those without IMD (41.3 pg/mL vs. 23.6 pg/mL) [21]. 

While the clinical utility of this difference may be a point of contention, Winther-Larsen et al. further illustrated the predictive value of NfL: NfL levels increased in all but one patient who developed IMD prior to presentation with symptoms that warranted brain-directed imaging, suggesting a role for NfL in cancer surveillance. Yet, the authors acknowledge transient NfL increases in three of these patients that could be attributed to receipt of chemotherapy or radiotherapy, likely leading to peripheral neuronal damage [22]. Lastly, patients with IMD and low serum NfL levels based on median NfL were found to have longer overall survival (OS) than patients with IMD and high serum NfL (HR 2.10, *p* = 0.01), though again, this association may be biased by neurotoxic therapies used in advanced systemic metastatic disease [19,22]. 

In summary, serum NfL levels appear to be specific to IMD and uninfluenced by disease stage. Combined with evidence that increases in NfL were observed prior to clinical detection of IMD and considering that NfL can easily be extracted from serum, continuous monitoring of NfL could be used in clinical practice to identify early IMD. Care should be taken as serum NfL levels may be falsely elevated in cancer patients with underlying neurologic disease or patients with recent unrecognized traumatic brain injury [15,16,17]. At present, it appears that appropriate candidates for NfL screening are those with primary cancers known to metastasize to the brain, who are not currently receiving chemotherapy or radiotherapy, and who have no history of recent neurologic injury or other neurologic comorbidity. Further evidence is required before NfL can be reliably employed as a diagnostic, predictive, or prognostic tool.

### 3.2. Glial Fibrillary Acidic Protein

Glial fibrillary acidic protein (GFAP) has also been considered as a potential serum marker for IMD. It is highly expressed in astrocytes and, like NfL, is frequently elevated in patients with neurodegenerative disease and traumatic brain injury [23,24]. In one cross-sectional study, patients with metastatic solid tumors and IMD were found to have higher serum GFAP levels compared to patients with metastatic cancer and no IMD, patients with no measurable disease but previous treatment for IMD, and healthy controls (555.7 pg/mL, 90.2 pg/mL, 97.1 pg/mL, and 74.5 pg/mL, respectively). Levels of GFAP were also elevated in patients with a primary CNS malignancy and differed between those with progressive and stable disease (2,092.1 pg/mL and 163.1 pg/mL, respectively) [20]. These findings were substantiated by Darlix and colleagues, who reported that among patients with breast cancer, those with IMD had elevated serum GFAP levels compared to those without IMD (median 292.7 pg/mL vs. 107.9 pg/mL, *p* < 0.001). Ubiquitin C-terminal hydrolase L1 and tau serum levels were also investigated and similarly showed significant elevations in patients with IMD compared to those without IMD. On multivariate analysis, however, high serum GFAP was the only factor independently associated with poor prognosis (HR 2.73, *p* = 0.002). An exploratory analysis on a subset of patients found increased serum GFAP levels three to four months prior to IMD diagnosis, mimicking those findings by Winther-Larsen et al. with respect to NfL [21]. These studies suggest that GFAP could act as a tumor marker for the early development of IMD. However, given that serum GFAP levels may be elevated in patients with neurodegenerative diseases or traumatic brain injury and no cut-offs have been defined to differentiate these pathologies, the utility of GFAP is currently limited [23,24].

The nascent state of studies examining proteins that arise through neurological damage in the setting of IMD precludes a definite conclusion on their suitability as IMD biomarkers. Additional studies, especially population-wide studies with healthy controls, patients with neurological disease, and patients with and without IMD, are necessary to clarify whether underlying neurological pathologies can lead to erroneous IMD diagnoses, define the utility of these markers in patient surveillance, and, if possible, establish serum level thresholds that facilitate the diagnosis of IMD.

## 4. Detection of Genetic Alterations Associated with IMD Using Liquid Biopsy

Our current best understanding of the molecular processes that underlie the development of metastatic disease centers on the concept of the epithelial-to-mesenchymal transition. This process involves cancerous epithelial cells transforming into mesenchymal-like cells, thereby facilitating their migration and ultimate dissemination to form distant metastasis [25,26]. During the metastatic process, tumor cells acquire metabolic, proliferative, and invasive signatures, which, if detected early, could predict metastases. Circulating tumor content, such as circulating tumor cells (CTC), circulating tumor DNA (ctDNA), extracellular vesicles, and microRNA (miRNA), may serve as markers for the genetic changes that drive the development of metastatic disease. Excitingly, circulating tumor content may be detectable using liquid biopsy, a minimally invasive technique that relies on the sampling of bodily fluids, such as serum and CSF [27]. An overview of current liquid biopsy candidates for IMD is given in Table 1. 

### 4.1. Circulating Tumor Cells 

Circulating tumor cells disseminated by primary or metastatic lesions have been identified in the peripheral blood of patients with IMD secondary to breast cancer, NSCLC, and melanoma [33,35,36,37,38]. Current research efforts have focused on the detection of CTCs as prognostic markers. For instance, in patients with non-metastatic or metastatic breast cancer, the presence of CTCs was found to be a significant indicator for shorter OS [55]. With regard to intracranial disease specifically, the LANDSCAPE trial found significantly higher 1-year OS (83.9% vs. 42.9%, *p* = 0.02) and CNS objective response rate (80% vs. 29%, *p* = 0.01) in patients with breast cancer who were CTC negative on day 21 of WBRT compared with those who were CTC positive on day 21 of the same treatment [35]. Despite low CTC detection rates in patients with IMD, Hanssen et al. also found CTC positivity rates, defined as ≥ 2 CTCs/7.5 mL blood, to be a strong predictor of OS in patients with IMD (HR 4.694, *p* = 0.004) [33]. 

Using CTCs as a biomarker for IMD has several major limitations. First, detection of CTCs is highly dependent on the method employed. Guedes de Casto et al., for example, were able to detect CTCs in 39/39 patients with melanoma before SRS using a method based on isolation by filtration [36]. By contrast, the majority of studies employ the CellSearch System (Veridex, Warren, NJ, USA), which relies on immunomagnetic isolation and separation of cells based on epithelial makers, and report CTC positivity rates in smaller proportions in cancer patients (ranging from 13% to 49% of patients being CTC positive) [32,33,34,35]. Second, detection of CTCs in peripheral blood is not specific to IMD, and, furthermore, detection rates can be lower in patients with IMD compared to other metastatic sites due to the lack of identifiable epithelial surface markers for isolation [36,56]. Hanssen et al., for example, reported even lower CTC positivity rates in patients with oligometastatic IMD based on a cut-off of ≥ 2 CTCs per 7.5 mL of blood (*n* = 34, 5.9% CTC positive) [33]. 

Genomic evidence suggests that there may be heterogeneity between CTCs due to specific metastatic organ sites, making the molecular characterization of CTCs one potential avenue to distinguish CTCs from distinct metastatic sites. Early evidence indicates that expression of CD44 and CD74 on CTCs, two proteins associated with the development of IMD, could be used to distinguish CTCs due to metastases to the brain from CTCs associated with other metastatic sites [37,38]. Considering the low overall detection rates of CTCs in patients with IMD using conventional assays, more in-depth investigations are required before CTCs can be reliably employed in a clinical setting for the prognosis and diagnosis of IMD. 

### 4.2. Circulating Tumor DNA

In contrast to CTCs, which are intact and viable cells, ctDNA is made up of fragments of nucleic acid that are present in circulation, likely as a consequence of tumor cell death [57]. Several proof-of-concept analyses have demonstrated that ctDNA can be isolated from plasma in patients with cancer [58,59,60,61]. Nevertheless, the detection of ctDNA in biofluids is challenging, as nucleic acids are often present at lower concentrations in biofluids than in cells or tissue, and when present, are often highly fragmented. The utility of ctDNA in the setting of IMD is further complicated by the fact that the blood–brain barrier may additionally restrict the release of ctDNA into the blood stream [28,58]. One study was able to detect plasma ctDNA in a cohort of 640 patients with a wide variety of primary cancers and demonstrated higher levels of detection in patients with metastatic compared to localized disease. However, ctDNA concentrations varied widely, and the ability to distinguish metastatic sites, in particular IMD, was not discussed [58]. In patients with melanoma, Lee et al. did not detect plasma ctDNA in any of the 13 patients with brain-only metastases and showed that ctDNA detectability was related to extracranial disease volume (*p* < 0.01), but not intracranial disease volume [62]. 

Alternatively, the data suggest that ctDNA is typically present in cerebrospinal fluid (CSF) in the setting of IMD, and that CSF ctDNA could be more representative of tumor genomic alterations and may more accurately be used to follow IMD progression compared to plasma ctDNA [43,63,64]. Our enthusiasm for this approach as a possible clinical tool is dampened by the need for lumbar puncture [64]. Beyond the relative contraindications to this procedure (including increased intracranial pressure and risk of cerebral herniation and coagulopathy, all of which are more likely to apply to patients with IMD), the use of ctDNA to detect IMD would require repeated lumbar punctures for continuous monitoring, increasing the risk for procedure-related complications and exposing patients to a more time-consuming and invasive form of testing [65]. Considering the limited amount of plasma ctDNA detectable in patients with IMD and the impracticability of obtaining CSF ctDNA, applications for ctDNA as a reliable biomarker at present are limited. 

### 4.3. DNA Methylation 

DNA methylation is an epigenetic process by which methyl groups are added to DNA; physiologically, DNA methylation is known to play a critical role in the regulation of gene expression [66]. Aberrant DNA methylation has been shown to be oncogenic, and many primary cancers present with stereotypic alterations in DNA methylation, making these patterns potential markers for detecting, monitoring, and characterizing disease [47,67]. In one multicenter study of 141 patients with primary breast cancer, for example, a high methylation index based on serum samples was independently associated with worse progression-free survival (HR 1.79, *p* = 0.002) and OS (HR 1.75, *p* = 0.003) [68]. 

In the context of primary brain malignancy, Nassiri et al. has demonstrated that DNA methylation signatures obtained from plasma samples could help distinguish healthy control samples from those of patients with primary brain malignancies as well as discriminate between extra-axial and intra-axial tumors with similar cell lines of origin [45]. To date, however, studies that have identified methylation patterns unique to IMD in patients with melanoma, NSCLC, and breast cancer have largely analyzed methylation patterns from tissue samples, as opposed to serum or other biofluids [48,49,69]. 

Analyses of DNA methylation could have practice-changing implications, as it does not necessitate the collection of CSF through a lumbar puncture and may be able to differentiate between types of intracranial tumors that have similar appearances on MRI. For now, the lack of data on plasma or serum methylation patterns in patients with IMD impedes any reliable conclusions to be drawn regarding its utility. 

### 4.4. Extracellular Vesicles 

Another component of liquid biopsy that has drawn attention is extracellular vesicles (including exosomes), which contain a diverse amount of protein, DNA, mRNA, miRNA, long non-coding RNA, and circular RNA, thus allowing them to capture cancer heterogeneity [51]. Expression profiles of microvesicle RNA captured in serum from patients with glioblastoma, for example, were distinct from RNA expression profiles in healthy controls [70]. 

Several in vitro studies have implicated cancer-associated extracellular vesicles in the pathogenesis of IMD. Notably, cancer cell-derived exosomes containing miR-105, miR-181c-5p, and long noncoding RNA GS1-600G8.5 have been shown to promote blood–brain barrier breakdown by altering endothelial cell permeability and tight junction protein regulation [71,72,73]. In contrast, miR-122-containing exosomes have been reported to regulate glucose metabolism in the metastatic microenvironment by suppressing glucose uptake and glucose catabolism in non-cancer cells [74]. The presence of these unique exosome signatures suggests that they have the potential as an approach to profile serum samples and detect metastatic events in the CNS. However, exosome analyses have proven to be a challenging tool to translate into clinical practice, as the profiling of exosomes requires their isolation from body fluids, which is technically challenging. Additionally, to date, no cancer-specific exosome markers have been identified [51,75]. These practical considerations must be addressed before exosome analyses can be feasibly incorporated into the clinical setting.

### 4.5. MicroRNAs

Several groups have identified circulating miRNAs with oncogenic or tumor suppressive properties in serum, plasma, and CSF, where they may exhibit cancer-specific expression patterns [76,77,78,79,80]. Both Teplyuk et al. and Nass et al. were able to distinguish primary brain malignancies from IMD secondary to solid tumors elsewhere in the body based on miRNA expression profiles derived from CSF [77,80]. However, as discussed previously, obtaining CSF for the purpose of biomarker analysis is both impractical and infeasible in active patient care. One study by Sato et al. demonstrated that the presence of specific miRNAs in serum from 78 patients with advanced breast cancer could significantly distinguish patients with IMD from those without IMD [53]. In patients with NSCLC, several different miRNAs identified in surgical tumor specimens have been suggested to be involved in mediating the progression towards IMD [81,82,83]. While these observations have important implications for understanding IMD tumorigenesis and identifying potential treatment targets, the lack of serum or plasma studies for the detection of circulation miRNA restricts its appropriateness as a biomarker. 

Taken together, liquid biopsy may allow the early detection of IMD in the future, but further evidence is required to clarify whether (1) analyses of liquid biopsy targets, chiefly CTCs, ctDNA, DNA methylation patterns, exosomes, or circulating miRNAs, can correctly differentiate IMD from other sites of metastases; and (2) less invasive techniques, such as blood sampling, can provide sufficient information on intracranial disease compared to CSF analyses. 

## 5. Biomarkers of Intracranial Metastases in the Context of Specific Primary Cancers

Given our advancing understanding of the molecular drivers and markers involved in the disease progression of NSCLC, breast cancer, and melanoma, and their predilection to metastasize to the brain, there is an increasing interest in uncovering biomarkers specific for the development of IMD in these three primary cancers. These disease-specific biomarkers are discussed below and summarized in Table 2. 

### 5.1. Non-Small Cell Lung Cancer

Brain metastases in patients with NSCLC are extremely common, with approximately 20% of patients harboring brain metastases at the time of diagnosis and as many as 50% developing IMD over the course of their disease [94,95]. Several studies have aimed to identify predictive markers of IMD risk in patients with NSCLC. For example, Saad et al. demonstrated that NSCLC patients with elevated Ki-67, VEGF-C, and Caspase-3 were at a significantly elevated risk of developing IMD (OR 12.2, *p* < 0.001; OR 14.6, *p* = 0.001; OR 43.0, *p* < 0.001). These differential expression patterns may help identify patients at higher risk for the development of IMD who would benefit from more frequent monitoring or could be used for the development of targeted therapies for IMD. 

Biomarker research to help with the prompt detection of IMD in patients with NSCLC is still at an early stage. One emerging biomarker is S100B, a calcium-binding protein that can inhibit apoptosis and promote cell proliferation [96,97]. Elevated levels of S100B are present in many conditions that damage the BBB, but it is of particular interest as a biomarker for IMD in patients with NSCLC [98]. Multiple studies have shown that levels of S100B are significantly higher in NSCLC patients with IMD compared to those without IMD [87,88,89,96]. For example, Pang et al. reported that NSCLC patients with IMD had a serum S100B concentration of 0.048 ± 0.0029 μg/L, while those without IMD had a serum S100B concentration of 0.015 ± 0.0160 μg/L (*p* ≤ 0.01) [89]. These differences are numerically small, but depending on the S100B threshold and technical corrections utilized, the sensitivity for the identification of IMD ranged from 89 to 94%, while specificity ranged from 43 to 93%, with an overall accuracy reported by Choi et al. of 62.5% [87,88]. Not all studies investigating S100B could confirm these findings: Kondrup et al. did not find any significant differences in S100B level between NSCLC patients with IMD and those without (0.049 μg/L vs. 0.044 μg/L, *p* = 0.852) [90]. In light of these conflicting findings, further studies with large patient cohorts will be required to validate the utility of S100B as a biomarker for IMD in patients with NSCLC. 

### 5.2. Breast Cancer

Much of the current literature on biomarkers in breast cancer IMD centers on tumor-specific mutations or gene expression changes. One potential avenue has been demonstrated in a proof-of-concept application of whole-exome sequencing using CSF-derived ctDNA to detect biomarkers associated with IMD. In this report, mutations in *TP53* and *PIK3CA*, as well as the overexpression of the known cancer-associated genes *ERBB2* and *cMYC*, were detected in a single patient [91]. Separately, Sato and colleagues compared serum miRNAs in breast cancer patients with IMD to those without IMD using high-sensitivity microarrays [53]. Two miRNAs, miR-4428 and miR-4480, were found to be significantly more common in the IMD cohort compared with the non-IMD cohort. The specificity and sensitivity were reported to be as high as 64.3% and 82.4% for miR-4428, and 76.5% and 71.4% for miR-4480, respectively, as biomarkers of IMD [53]. Though not yet validated in large population-based studies, these results suggest that the detection of IMD in breast cancer patients without surveillance imaging or biopsy may be feasible. More work on the use of serum or CSF miRNAs for the detection of IMD will be necessary to establish the clinical utility of such assays.

### 5.3. Melanoma

Several serum markers associated with melanoma survival or response to therapy, including lactate dehydrogenase and CD73, have been described, but few studies have successfully identified and validated serum or CSF markers of melanoma-associated IMD, which occurs in over 50% of melanoma patients [99,100,101]. The use of cytopathology to detect whole circulating melanoma cells in CSF has been described in the diagnosis of leptomeningeal disease; however, the application of this technique may be limited by its specificity, as patients without circulating melanoma cells may have IMD [102]. 

Early work by Hoon and colleagues demonstrated that reverse transcriptase-polymerase chain reaction (RT-PCR) assays in CSF may be more sensitive than cytopathology. Melanoma-associated mRNA markers (MAGE-3, MART-1, and tyrosinase) could be detected by RT-PCR in approximately 50% of patients with melanoma who developed IMD during the follow-up period [92]. Though these analyses are limited by the necessity to perform lumbar punctures for the collection of CSF, RT-PCR may play an emerging role in the diagnosis of IMD secondary to melanoma.

Rather than focus on a single marker, Lok and colleagues described cytokine and chemokine alterations in CSF obtained from melanoma patients with IMD [93]. Compared to CSF obtained from healthy controls, these samples contained reduced levels of CCL22, IL-1α, IL-4, and IL-5, as well as increased levels of CXCL10, CCL4, CCL17, and IL-8 [93]. Furthermore, patients with elevations in CXCL10, CCL4, and CCL17 and reductions in CCL22, IL-1α, IL-4, and IL-5 demonstrated shortened time between melanoma diagnosis and the development of IMD. Further, patients with elevated CCL17 and IL-6 and reduced CCL22, IL-1α, IL-1β, IL-4, IL-5, and IL-6 exhibited poor OS following IMD diagnosis [93]. Taken together, CSF cytokine and chemokine alterations may be a sensitive and specific marker for IMD diagnosis and prognosis in patients with melanoma.

## 6. Imaging Biomarkers

MRI remains the gold standard for intracranial surveillance and the detection of IMD, with efforts currently underway to extend its utility and address its shortfalls [12,103]. Markers on MR perfusion imaging, for example, can predict response to treatment with SRS or WBRT [104,105,106,107]. Similarly, diffusion MRI is being investigated as an imaging biomarker of tumor aggressiveness and early response to therapy [108,109]. Although these techniques depend on the skill of the radiologist, in the future, these markers could be incorporated into prognostic models identifying patients at higher risk for developing recurrence in the brain [110]. 

Another avenue for biomarker imaging is radiomics, a field aimed at describing tumor heterogeneity using conventional MRI based on the assumption that radiographic findings represent underlying physiologic changes [111]. Using radiomics, investigators have defined structural and textural features that may serve as predictors of survival and could be used to identify patients who would benefit from more frequent follow up [112,113]. Currently, however, the field has only limited insight into the biological underpinnings of these findings, limiting the translation of radiomic findings into clinical practice. Presently, attempts to correlate imaging with biology through validation with genetic and histologic data are underway [114]. 

In addition to MRI and MRI radiomics, some authors have explored the use of positron emission tomography (PET) for the diagnosis of IMD. Unfortunately, these studies have largely concluded that the utility of PET in the brain is limited. Krüger et al. demonstrated that ^18^F-2-fluoro-2-deoxy-D-glucose (FDG) PET has a sensitivity of only 27% for the detection of IMD in patients with lung cancer [115]. A larger meta-analysis of similar work showed a cumulative sensitivity of just 21% using PET in comparison to 77% when using MRI for the diagnosis of IMD [116]. The poor sensitivity of FDG PET, which relies on glucose uptake and metabolism, has been largely attributed to the fact that the brain is an inherently metabolically active organ, making it difficult to distinguish malignant lesions from the neuronal background [117]. Of note, amino acid-based *O*-(2-[^18^F]fluoroethyl)-L-tyrosine (FET) PET appears to be more sensitive than the traditional FDG PET, with a quoted sensitivity approaching 90%, but less than that of MRI (100%). In the same study, FET-PET was shown to have poor sensitivity to detect brain metastases smaller than 1 cm. Taken together, the literature on PET imaging and IMD does not currently support the routine, clinical use of PET in the diagnosis of IMD [118]. 

## 7. Conclusions and Future Directions

The development of IMD poses a significant clinical challenge in patients with metastatic cancer. Although advances in systemic treatments have prolonged survival, the development of IMD continues to exert a profound adverse effect on patient survival and quality of life, making the early detection of IMD crucial for the modification of therapies to target IMD. Conventional methods for the detection of IMD are typically prompted by the development of neurological symptoms, and as such, fail to identify patients in the early stages of disease. New biomarkers for the early detection of IMD and prognostic evaluation are under investigation, including markers of early brain damage, genetic alterations specific to IMD detectable via liquid biopsy, and imaging biomarkers. Several small-scale studies have also investigated biomarkers specific to IMD in NSCLC, breast cancer, and melanoma skin cancer, the primary cancers most implicated in the development of IMD. While this work remains in its early stages, its possible value to clinical care merits further investigations to establish the utility of these biomarkers in real-world clinical settings.

## Figures and Tables

**Table 1:** Candidate markers for the detection of IMD via liquid biopsy [27].

Biomarker	Advantages	Disadvantages
CTC	Can be obtained from peripheral blood and is thus minimally invasive [28] Can be used for cytologic, genome, proteome, and transcriptome analyses Strong evidence for role as prognostic marker in systemic disease [29,30,31]	Limited presence in the blood stream [32,33,34,35] Varying levels of detection depending on the quantification method [32,33,34,35,36] Limited ability to distinguish IMD from other distant metastatic sites [37,38]
ctDNA	Established role in the diagnosis, treatment, and management of systemic malignancies [39] Next-generation sequencing (whole genome/exome) enables detection of novel mutations and tumor heterogeneity [40,41] Droplet digital PCR and similar advanced amplification techniques enable sensitive detection of common and known polymorphisms associated with cancer [42]	Unreliable as a plasma marker and requires lumbar puncture for CSF sampling [43] Limited ability to differentiate between ctDNA from tumor cells and ctDNA from native cells [44]
DNA methylation patterns	DNA methylation patterns can be used to differentiate IMD from primary brain tumors [45] DNA methylation patterns are associated with gene expression changes in malignancy [46,47]	Detection of DNA methylation in serum has only been reliably reported from analysis of solid tumors [48,49]
Extracellular vesicles	Contain non-coding RNAs, including miRNA, associated with systemic disease status [50] Exosome cargo is diverse and can, therefore, capture cancer cell complexity [51]	Detection of vesicles remains challenging due to lack of established methodologies for extraction from body fluids [50,51] Studies on extracellular vesicles mostly limited to in vitro descriptions [50]
miRNA	Established role in the diagnosis, treatment, and management of systemic malignancies [52] Microarray enables sensitive detection of known miRNAs associated with cancer [53] miRNA detectable in body fluids, including serum and CSF [52,53]	Further research needed to improve the sensitivity and specificity of miRNA assays in the detection of IMD [52,54]

CTC: circulating tumor cell; CSF: cerebrospinal fluid; ctDNA: circulating tumor DNA; IMD: intracranial metastatic disease; miRNA: microRNA; PCR: polymerase chain reaction.

**Table 2:** Summary of studies investigating candidate markers for the detection of IMD specific to NSCLC, breast cancer, and melanoma skin cancer.

	Study	Participant Numbers	Biomarkers of Interest (Sample Type)	Findings
NSCLC	Saad et al. [84]	IMD: 21, no IMD: 33	Ki-67, caspase-3, VEGF-A, VEGF-C, E-cadherin, EGFR (tissue)	Significantly increased risk of developing IMD associated with: high Ki-67, low caspase-3, high VEGF-C, low E-cadherin No significant risk associated with: VEGF-A and EGFR
	Gomez-Roca et al. [85]	IMD: 9, no IMD: 40	EGFR, ERCC1, VEGFR, Ki-67 (tissue)	Significantly increased expression in IMD samples compared to samples from the primary site: ERCC1 Decreased levels of EGFR expression in metastases, but no significant difference in different metastatic sites No significant differences: VEGFR, Ki-67
	Grinberg-Rashi et al. [86]	IMD: 25, no IMD: 82	KIFC1, KIFC2, KIG14, CCNB2, SIL, TNPO1, LMNB1; CDH2, SGNE1, FALZ, ADAM8, SPP1 (tissue)	Positive predictive effect: CDH2, KIFC1 Negative predictive effect: FALZ No significant effect observed for any of the other genes Generated predictive score based on expression of: CDH2, KIFC1, FALZ
	Chen et al. [87]	NSCLC+ IMD: 100, NSCLC no IMD: 50, CVD: 50	S100B protein S100B antibody (serum)	S100B significantly elevated in patients with IMD compared with patients without IMD or CVD No significant differences between patients without IMD and CVD No significant difference in levels of S100B antibody Patients with IMD and high levels of S100B had significantly shorted OS and PFS compared to patients with IMD and low levels of S100B S100B sensitivity: 94%, specificity: 93% (cut-off: 0.014 ng/mL)
	Choi et al. [88]	IMD: 18, no IMD: 110	S100B protein S100B antibody (serum)	S100B protein sensitivity: 89%, specificity: 43%, accuracy: 51%, (cut-off: 0.058 ng/mL) S100B protein + autoantibody - sensitivity: 89%, specificity: 58%, accuracy: 62.5% (antibody threshold: < 2.00 absorbance units)
	Pang et al. [89]	IMD: 15, no IMD: 15	S100B protein (serum)	S100B significantly higher in IMD group Infection of cells with full-length S100B expression vectors significantly promoted cell proliferation and inhibited apoptosis
	Kondrup et al. [90]	IMD: 22, no IMD: 50	S100B protein (serum)	No significant difference in S100B
Breast cancer	Siravegna et al. [91]	Single patient	ctDNA (plasma and CSF)	Comparison of pre-treatment and post-treatment samples: reduction in plasma ERBB2, tp53, and PIK3CA consistent with extra-cranial disease control but not CSF-derived tp53 and PIK3CA consistent with non-response Plasma ERBB2 amplification, tp53 and PIK3CA mutations were detected at the time of CNS progression
	Sato et al. [53]	IMD: 51, no IMD: 28	miRNA (serum)	miR-4428 and miR-4480 could distinguish IMD from non-IMD (greater than 2-fold change between the groups, p<0.001) miR-4428 sensitivity: 82.4%, specificity: 64.3% miR-4480 sensitivity: 76.5%, specificity: 71.4%
Melanoma skin cancer	Hoon et al. [92]	total: 37	MAGE, MART-1, tyrosinase (CSF)	MART-1 and/or MAGE-3 were positive predictive markers for the development of IMD RT-PCR could detect approximately 50% of patients who developed IMD during a 4-year follow up period based on only a single time point
	Lok et al. [93]	IMD: 22, healthy control: 5	Cytokines and chemokines (CSF)	Cluster analysis revealed that suppression of IL1α, IL4, IL5, and CCL22, with concomitant elevation of CXCL10, CCL4, and CCL17 correlated with more aggressive IMD (time to IMD and survival outcomes)

CCNB2: cyclin B2; CDH2: N-cadherin; CSF: cerebrospinal fluid; CVD: cerebrovascular disease; EGFR: epidermal growth factor receptor; ERCC1: excision repair cross-complementing; FALZ: fetal Alzheimer antigen; IMD: intracranial metastatic disease; KIFC1: kinesin family member C1; LMNB1: lamin B1; NSCLC: non-small cell lung cancer; OS: overall survival; PFS: progression free survival; SIL: SCL-TAL1 interrupting locus; SGNE1: secretogranin V; TNPO1: transportin 1; VEGFR: vascular endothelial growth factor receptor.
